# ZEB1 enhances Warburg effect to facilitate tumorigenesis and metastasis of HCC by transcriptionally activating PFKM

**DOI:** 10.7150/thno.56490

**Published:** 2021-04-03

**Authors:** Yanming Zhou, Furong Lin, Tao Wan, Ai Chen, Huihui Wang, Bin Jiang, Wentao Zhao, Shan Liao, Shijie Wang, Guannan Li, Zhenzhen Xu, Jinyang Wang, Jia Zhang, Huanhuan Ma, Donghai Lin, Qinxi Li

**Affiliations:** 1 The State Key Laboratory of Cellular Stress Biology, Innovation Center for Cell Signaling Network, School of Life Sciences, Xiamen University, Xiamen, Fujian 361102, China.; 2 Department of Hepatobiliary & Pancreatovascular Surgery, The First Affiliated Hospital of Xiamen University, Xiamen, Fujian 361003, China.; 3 Department of Chemical Biology, College of Chemistry and Chemical Engineering, Xiamen University, Xiamen, Fujian 361005, China.

**Keywords:** ZEB1, glycolysis, PFKM, non-classic binding, intrahepatic metastasis

## Abstract

Metabolic reprogramming, especially Warburg effect, is a key event in tumor initiation and progression. ZEB1 plays a vital role in metastasis of various cancers. We previously found that ZEB1 was excessively expressed in hepatocellular carcinoma (HCC) and its high expression was closely correlated with metastasis and recurrence of HCC. We want to know whether glycolytic enzymes are regulated by ZEB1 and contribute to carcinogenesis and metastasis of HCC.

**Methods:** To explore whether ZEB1 could enhance glycolysis in HCC, we knocked down ZEB1 by short hairpin RNA (shRNA) in MHCC-97H and HCC-LM3 cells and performed glucose uptake, lactate production, ECAR and OCR assays. To investigate how ZEB1 enhances glycolysis, the protein levels of glycolytic enzymes were detected in the same cell lines using Western blot. The regulatory effect of ZEB1 on PFKM mRNA level was confirmed by RT-qPCR, luciferase report assay and ChIP assay. In order to assess the role of ZEB1-PFKM axis in cell proliferation, cell counting and CCK-8 assays were performed in MHCC-97H and HCC-LM3 cell lines knocked down for ZEB1 and further re-expressed for either ZEB1 or PFKM or not. To explored whether the ZEB1-PFKM axis also functions in HCC cell migration, invasion and metastasis, the same MHCC-97H and HCC-LM3 cell lines were performed for wound healing assays, transwell assays and colony formation assays, meanwhile, MHCC-97H cell lines were performed for orthotopic liver transplantation assays. Finally, the expression of ZEB1 and PFKM were examined in human liver cancer specimens and non-tumorous liver tissues using immunohistochemical and Western blot.

**Results:** We found that ZEB1 transcriptionally upregulates the expression of the muscle isoform of phosphofructokinase-1 (PFKM), a rate-limiting enzyme in glycolysis. Intriguingly, a non-classic ZEB1-binding sequence in the promoter region of PFKM was identified through which ZEB1 directly activates the transcription of PFKM. Silencing of ZEB1 in MHCC-97H and HCC-LM3 cell leads to impaired PFKM expression, glycolysis, proliferation and invasion, and such impairments are rescued by exogenous expression of PFKM. Importantly, *in-situ* HCC xenograft assays and studies from TCGA database demonstrate that ZEB1-PFKM axis is crucial for carcinogenesis and metastasis of HCC.

**Conclusions:** Our study reveals a novel mechanism of ZEB1 in promoting HCC by activating the transcription of PFKM, establishing the direct link of ZEB1 to the promotion of glycolysis and Warburg effect and suggesting that inhibition of ZEB1 transcriptional activity toward PFKM may be a potential therapeutic strategy for HCC.

## Introduction

Hepatocellular carcinoma (HCC) is one of the most frequently occurring cancer type worldwide and is the fourth common cause of cancer death 1. Much is known about the development and cause of HCC. Approximate 70%-90% of HCC patients develop from chronic hepatitis, and diabetes is also an independent risk factor of HCC 2, 3. Up to now, the basal therapeutic choice for HCC patients without cirrhosis and multifocality is still hepatic resection due to the lack of effective chemotherapy drugs resulting from the poor understand of the molecular mechanism underlying pathogenesis of HCC 3. Unfortunately, more than 70% hepato-resected patients appear post-resection recurrence in 5 years with poor histological differentiation, microvascular, multifocal disease and satellites 3. Invasion, metastasis and recurrence are responsible for the majority of HCC-caused death cases. Therefore, to find effective targets for HCC therapy by uncovering the molecular mechanism underlying HCC occurrence and metastasis is of great significance for improving survival rate of HCC patients.

Zinc finger E-box-binding homeobox 1 (ZEB1) is a member of ZEB family and generally identified as a transcription factor. ZEB1 contains two zinc-finger clusters at the C- and N-terminal regions respectively which can bind to E2-box-like CACCT(G) sequences in the promoter region of target genes 4. ZEB1 promotes epithelial-mesenchymal transition (EMT) by suppressing the expression of cell adhesion molecule E-cadherin, a critical transmembrane protein in maintaining the epithelial phenotype 5. High expression of ZEB1 has been observed in intrahepatic cholangiocarcinoma, colorectal cancer, gastric carcinoma, pancreatic cancer, esophageal squamous cell carcinoma and oral cavity carcinoma 6, suggesting that ZEB1 may contribute to the progression of these tumors. Moreover, elevated expression of ZEB1 seems to predict worse overall survival in cancer patients 6. We and others have showed that ZEB1 was highly expressed in HCC and its high expression was correlated with advanced TNM stage, tumor size, intrahepatic metastasis, vascular invasion, and frequent early recurrence 7. Depletion of ZEB1 in pancreatic cancer mice model (induced by mutant *Kras* and *p53*) strongly reduced malignancy and metastasis of pancreatic cancer 8. These data suggest that ZEB1 plays an important role in tumor progression.

Most cancer cells prefer to metabolize glucose and generate ATP principally through aerobic glycolysis even under sufficient oxygen condition. This phenomenon was first described by Otto Heinrich Warburg and is the most remarkable characteristic of cancer metabolism 9. Recent studies have shown that glycolysis may support cancer metastasis by lowering the tumor microenvironment pH value, contributing to ATP generation and impacting signaling pathways 10. Inhibition of glycolysis enzyme, such as hexokinase 2 (HK2) 11-13, 6-phosphofructo-2-kinase/fructose-2, 6-bisphosphatase 3 (PFKFB3) 14 and pyruvate kinase M2 (PKM2) 15, results in reduced cancer cell invasion and metastasis, indicating the critical role of glycolysis in these biological events. As a transcriptional factor ZEB1 is an important driver of EMT and tumor progression. However, whether ZEB1 can upregulate glycolytic enzymes and consequently contribute to tumorigenesis and metastasis is largely unknown. Based on our previous study on ZEB1 in HCC, we investigated its regulatory effect on glycolytic enzymes in HCC cell lines MHCC-97H and HCC-LM3. Our results demonstrate that ZEB1 can induce glycolysis via upregulating PFKM, a rate-limiting enzyme in glycolysis. Mechanistically, ZEB1 directly binds to a non-classic ZEB1-binding sequence on the promoter region of PFKM and therefore activates its transcription. Importantly such glycolysis-promoting function of ZEB1 contributes to proliferation and intrahepatic metastasis of HCC to a great extent.

## Materials and methods

### HCC sample

This study was approved by the clinical research ethics committee of the First Affiliated Hospital of Xiamen University (Xiamen, Fujian, China). Before tissue acquisition, written informed consent was obtained from each patient according to the policies of the committee. 20 HCC tissues and paired non-tumor tissues were used for Western blot and immunohistochemical (IHC) staining.

### Plasmids construction

Full length human ZEB1 (gene ID: 23383) and PFKM (gene ID: 12981) cDNAs were obtained from Dr. Jiahuai Han (State Key Laboratory of Cellular Stress Biology, School of Life Sciences, Xiamen University), and inserted into lentivirus vector pBoBi. PLL3.7-Neo lentivirus vector was used to express shRNAs for knockdown of ZEB1 and PFKM. The synonymous point mutations residing in the shZEB1#2 target sequence of Flag-tagged ZEB1 expression plasmid were created for RNAi resistance. The primers and siRNA target sequence are listed in Table S2.

### Cell culture and stable cell line construction

MHCC-97H and HCC-LM3 (high metastatic potential) were obtained from the Liver Cancer Institute, Fudan University, Shanghai, China. HUH-7, PLC/PRF/5, Hep3B, HepG2, YY8103, SNU-449 and LO2 were obtained from ATCC. Cells were cultured in Dulbecco modified Eagle's medium (DMEM) supplemented with 10% fetal bovine serum (GEMINI, A24G00J) in a 37 °C humid incubator containing 5% CO_2_ and examined negative for mycoplasma infection using PCR-based Mycoplasma Detection Kit (Sigma, MP0035-1KT). To obtain a stable ZEB1 knockdown MHCC-97H cell line, lentiviral particles were generated by co-transfecting shZEB1 plasmid with packaging plasmid mix (REV: VSVG: PMDL=2:3:5) using turbofect transfection reagent (Fermentas, R0532) in HEK-293T cells and harvested 36-48 h later. MHCC-97H cells were then infected with a proper amount of virus in the presence of polybrene (Merck Millipore, TR-1003-G) at a final concentration of 10 µg/mL. After 48 h of infection, cells were selected with G418 (Sigma, PP2374-1KT) to obtain stable cell line for follow-up experiments. The similar operation process was conducted for the construction of MHCC-97H and HCC-LM3 cell lines re-expressing ZEB1 or PFKM and HUH-7 and Hep3B cell lines overexpressing ZEB1.

### Glucose uptake and lactate production assay

For glucose uptake, cells were seeded into 6-well plates for 24 h. When the confluence reached 80%, cells were treated with low glucose (5 mM) medium for 4 h and no glucose medium for 30 min and no glucose medium containing 2.5 µg/mL 2NBDG for 30 min. Then cells were washed with PBS three times and collected to measure fluorescence value by flow cytometer. Lactate production were measured using lactate colorimetric/fluorometric assay kit (Biovision, K607-100) according to the manufacturer's protocol. Firstly, the cells were seeded into 6-well plates and were cultured with complete medium for 24 h. Then medium was exchanged to fresh medium containing 5 mM glucose and were incubated for 15 h. Finally, medium was collected for measurement of lactate level, cells were lysed for normalization and Western blot.

### Western blot

Cells cultured for Western blot were harvested in a lysis buffer (20 mM Tris-HCl, pH 7.4, 150 mM NaCl, 1 mM EDTA, 1 mM EGTA, 1% Triton X-100, 2.5 mM sodium pyrophosphate, 1 mM β-glycerolphosphate, 1 mM sodium orthovanadate, 1 µg/mL leupeptin, 1 mM phenylmethyl sulfonyl fluoride). Cell lysate was sonicated 5 times for 2 s each, then centrifuged at 15,000 g for 30 min at 4 °C to obtain the supernatant. Proteins in total cell lysate were separated in 10% SDS-PAGE and transferred to PVDF membrane (Roche). The membrane was blocked with 5% nonfat milk in TBST buffer (20 mM Tris-HCl, 150 mM NaCl and 0.1% Tween-20, pH 7.5) for 1 h and incubated with corresponding antibodies (antibody related information are listed in Table S1). Proteins were visualized by enhanced chemo luminescence (ECL) detection reagents. For density quantization, Image-Pro Plus 6.0 was used to measure the IOD, color selection was based on color cube.

### Seahorse assay

An XF96 extracellular flux analyzer (Seahorse Bioscience) was used to determine the effects on glycolysis and oxidation phosphorylation of ZEB1 knockdown in MHCC 97H and ZEB1 overexpressed HUH-7 cells. Cells were seeded in an XF96 cell culture plate to reach 90% confluence, which were seeded at 12,000 cells/well. Right before the assays, these cells were changed from a culture medium to an assay medium and incubated for 1 h at 37 °C. After baseline measurements, various chemicals prepared in the assay medium were sequentially injected into each well and subjected to the measurement of ECAR or OCR respectively. ECAR is reported as mpH/min and normalized to protein concentration, OCR is reported as pmol/min and normalized to protein concentration. After the completion of the experiment, cells were immediately trypsinized and normalized individual well rate data to protein concentration.

### LC-MS

Briefly, MHCC-97H cells cultured to around 80% confluence were washed in pre-cold PBS 3 times, quenched in 1 mL cold (-80 °C) 80% methanol (methanol/water, v/v) and were detached from the culture dish using a cell scraper. Quenched cells were centrifuged at 12,000 g for 15 min at 4 °C, and 0.8 mL supernatant was dried under nitrogen gas and dissolved in 100 μL aqueous acetonitrile water. The mixture was centrifuged at 12,000 g for 15 min and analyzed by LC-MS within 24 h. Sample separation and analysis were performed on a Waters Acquity TM BEH C18 column (2.1 mm×50 mm, particle size of 1.7 μm) using a gradient of buffer A [10 mM tributylamine, 15 mM acetic acid, 3% (v/v) methanol in water] and buffer B (methanol) and using multiple reaction monitoring (MRM) transitions.

### Intracellular ATP assay

ATP level was measured using the ATP assay kit (Beyotime, S0026) according to the manufacturer's instructions. In brief, cells were seeded in 12-well plate for 24 h. Then cells were harvested by adding 200 μL lysis buffer and centrifuged at 12,000 g for 5 min at 4 °C. The supernatant was mixed with detection solution and then analyze for ATP concentration with a Multiscan Spectrum (Luminescence).

### RT-qPCR

Total RNA was extracted using Trizol reagent (Invitrogen, 15596026) according to the manufacturer's instruction. The first strand cDNA was synthesized by Revert Aid Reverse Transcriptase Kit (Thermo Scientific, B300100-0010) with Oligo (dT) primers. ACTB (Beta-actin) was used as endogenous control and gene expression levels were determined using pre-validated SYBR Green Power Master Mix (Applied Biosystems, 4367659). Amplification levels were detected using the ABI PRISM 7900HT Sequence Detection System (Applied Biosystems). All mRNA transcript levels were showed as the ratio of ACTB. The primers for RT-qPCR are listed in Table S2.

### Immunohistochemical staining

For immunohistochemistry (IHC), 5 μm thick sections were deparaffinized and rehydrated using xylene and a graded series of ethanol. Antigen retrieval was performed in 10 mM sodium citrate buffer (pH 6.0), which was microwaved at 100 °C for 20 min. Sections were incubated with endogenous peroxide scavenging agents and then blocked using 5% normal horse serum, followed by incubation with anti-ZEB1, anti-PFKM and anti-PCNA antibody (antibody related information are listed in Table S1) overnight at 4 °C. Colors were developed with an Optiview DAB IHC detection kit (Abcam, ab236466). For calculation of IHC score, photographs of four randomly selected fields (magnification, ×200) from each slide were got and Image-Pro Plus 6.0 software was used to measure the sum of IOD (integrated option density) of each photograph. The final IHC score was presented as the average of IODs from four pictures and normalized to that of corresponding adjacent normal sample. 7 pairs of human primary HCC samples and corresponding adjacent normal samples were used for IHC.

### Luciferase reporter assay

The PFKM genomic fragment (Chr: 12q13.11 48105253-48146404) containing promoter region -2000+1 was amplified and cloned into pGL2-basic vector. The reporter plasmids were co-transfected with pBoBi-ZEB1 into HEK-293T cell. 24 h after transfection, the luciferase activity was determined by Luciferase Assay Kit. The luciferase activity was normalized by protein concentration. The primers for PCR are listed in Table S2.

### ChIP assay

For chromatin immunoprecipitation (ChIP) experiment, one 150 mm dish of MHCC-97H cells were treated with 1% formaldehyde at room temperature for 10 min to crosslink chromatin proteins to DNA. Then a final concentration of 0.125 M glycine was added to the dish for 5 min to terminate crosslink. Cells were lysed in lysis Buffer (50 mM Tris-HCl pH 8.0, 0.5 mM EDTA, 1% SDS and protease inhibitors) and the resulting lysate was sonicated to break chromatin into fragments with an average length of 300~500 bp, followed by immunoprecipitation with anti-ZEB1 antibody and purification of immunoprecipitated DNA fragments. PCR was then performed with the following primers to determine whether ZEB1 binds to these genes. The PCR samples were separated and stained by electrophoresis in the 2% agarose gel contain with ethidium bromide. The primers for PCR are listed in Table S2.

### *In vitro* cell behavior assays

Cell proliferation was assessed by cell counting assay and enhanced cell counting kit-8 (CCK-8) assay. For cell counting, 1×10^5^ cells were seeded into 6-well plate and grown in complete DMEM medium at 37 °C with 5% CO_2_ for designed time. Then cells were counted by Count Star. Experiment of each group was performed three times. For CCK-8 assay, 2×10^3^ cells were seeded into 96-well plates and incubated in complete DMEM medium at 37 °C in a humid incubator containing 5% CO_2_ for designed time. Cell viability was determined using CCK-8 kit. All tests were performed three times in quadruplication.

For wound healing assay**,** cells were seeded to 6-well plate and cultured to reach 90% confluence. Then the cell monolayer was scratched gently with a 200 μL pipette tip, washed twice with PBS to remove detached cells, and the remaining cells were incubated in medium without FBS. The scratch areas were photographed at 0 h and 48 h. Experiment of each group was performed in triplicate.

Cell invasion assay was performed using transwell chambers. 2×10^5^ cells were seeded into the upper transwell chamber (poly carbonate filter with 8 μm pores, coated with 200 μg/mL Matrigel) containing 200 μL serum-free medium. The bottom chamber was supplemented with 800 μL complete medium as chemo attractant. After 48 h incubation, noninvasive cells were removed with a cotton swab from the upper surface of the filters and invasive cells on the lower surface of the filters were fixed and stained with 0.5% crystal violet at room temperature for 10 min. The numbers of cells were counted in five random microscopic fields. Experiment of each group was completed in biological triplicate.

Tumorigenic ability was evaluated by colony formation assays. In brief, 0.5 mL complete DMEM medium containing 0.35% agar was poured into 12-well plate at first and acted as bottom layer to segregate the cells from touching with the surface of plate bottom. After solidifying, a top layer medium containing 0.35% agar and 1000 cells was poured. The plates were placed in the incubator and the medium was changed every 3 days for 24 days. Then the colonies were fixed with 4% paraformaldehyde in PBS and stained with 0.01% crystal violet solution. The colonies larger than 100 μm were counted per well. Experiments of every groups were completed in biological triplicate.

### Orthotopic liver transplantation assay

All animal experimental protocols were approved by the institutional Animal Care and Use Committee at Xiamen University. All the mice were BALB/C nude mice and five 6-week-old male mice were used for experiments. Briefly, 2.0×10^6^ MHCC-97H cells suspended in 50 μL PBS were injected into the same site of the left liver lobe of nude mice to develop orthotopic liver tumor. 6 weeks after injection, the mice were euthanized, livers were isolated from each group of mice and the weight and size of primary tumor in the injection site of each liver were determined to indicate the tumorigenic ability of each cell lines. Simultaneously, the number of intrahepatically metastatic tumors excluding the primary tumor in each liver was counted to reflect the metastatic capability of injected cell lines. Tumor volume was calculated as follows: tumor volume [mm^3^] = (length [mm]) × (width [mm])^2^ × 0.5.

### Tumor database analysis

The gene expression data of HCC patient samples are available in the Oncomine database (https://www.oncomine.org). The survival data were got from the Kaplan Meier Plotter (http://kmplot.com/analysis/).

### Statistical analysis

Data were expressed as mean ± SD/SEM. In order to identify significant differences in the data, statistical analyses were performed with one-way ANOVA or two-tailed unpaired Student's *t*-test. *P < 0.05, **P < 0.01, ***P < 0.001, N.S.: no significant difference (*P* ≥ 0.05).

## Results

### ZEB1 enhances glycolysis and promotes the Warburg effect in HCC cell lines

**Figure 1:**
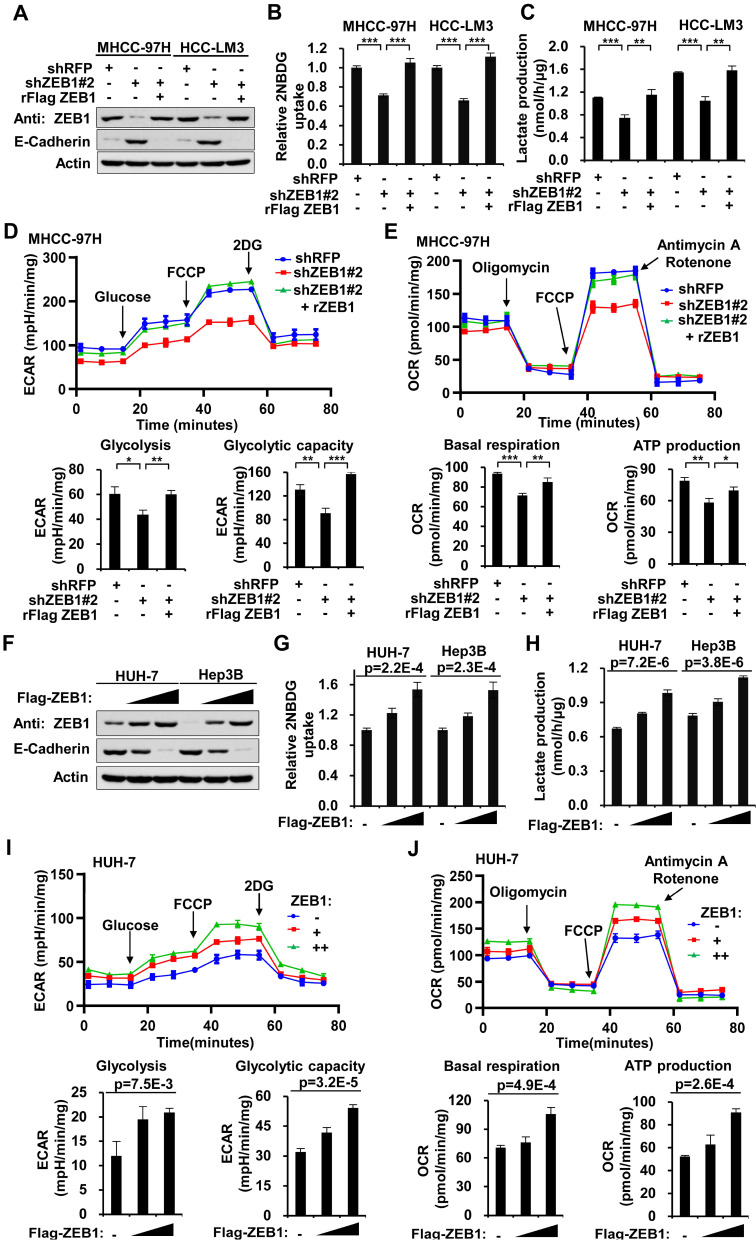
** High expression of ZEB1 enhances glycolysis. (A)** Western blot analysis of the ZEB1 protein level in MHCC-97H and HCC-LM3 cell lines with knockdown and further re-expression of ZEB1. E-Cadherin protein levels were detected as a positive control for ZEB1 knockdown. **(B)** Glucose uptake and **(C)** lactate production were measured in MHCC-97H and HCC-LM3 cell lines. **(D)** MHCC-97H cells used in (A) were detected for extracellular acidification rate (ECAR) as an indicator for deduced glycolysis flux and glycolytic capacity. **(E)** The oxygen consumption rate (OCR) was detected as an indicator for oxidative phosphorylation (OXPHOS) and deduced levels of basal respiration and ATP production. **(F)** HUH-7 and Hep3B cells were expressed for increasing doses of ZEB1, followed by detection of ZEB1 protein level, **(G)** glucose uptake and **(H)** lactate production.** (I, J)** HUH-7 cells used in (F) were detected for ECAR as an indicator of glycolysis flux and glycolytic capacity **(I)**, and OCR as an indicator of OXPHOS and deduced levels of basal respiration and ATP production **(J)**. The data in Figure 1 except B and F are shown as means±SD of three independent experiments (*P<0.05, **P<0.01, ***P<0.001). The Statistical analyses in (B-E) and in (G-J) are performed using unpaired Student's *t* test and one-way ANOVA, individually.

**Figure 2:**
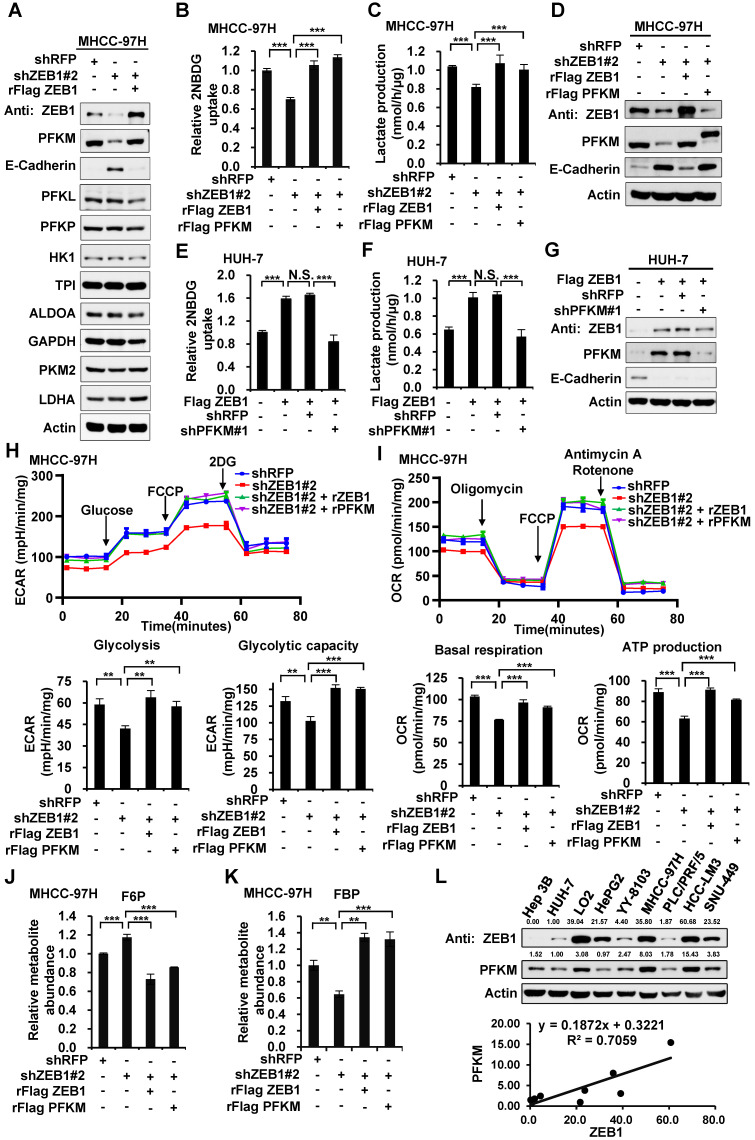
** ZEB1 stimulates glycolysis by upregulating PFKM**. **(A)** Immunoblot of glycolysis enzymes in ZEB1 knockdown and re-expression MHCC-97H cell lines. **(B-D)** MHCC-97H cells were knocked down for ZEB1 and further expressed for exogenous ZEB1 or PFKM, followed by determination of glucose uptake (B), lactate production (C) and expression levels of indicated proteins (D).** (E-G)** HUH-7 cells with low endogenous expression of ZEB1 was overexpressed for Flag-tagged ZEB1 and further knocked down for PFKM, followed by measurement of glucose uptake (E), lactate production (F) and protein levels (G). **(H)** MHCC-97H cells used in (B) were detected for ECAR to indicate glycolysis flux and glycolytic capacity. **(I)** The OCR was detected to indicate basal respiration and ATP production. **(J-K)** Relative abundances of F6P (J) and FBP (K) in the same MHCC-97H cell lines as in (B) were measured using LC-MS. **(L)** The protein levels of ZEB1 and PFKM in a series of HCC cell lines were detected (upper panel) and analyzed for their correlation (lower panel). The data in (B-C, E-F and H-K) are shown as means±SD of three independent experiments and analyzed using Student's *t* test (**P<0.01, ***P<0.001, N.S.: *P* ≥ 0.05).

To explore whether ZEB1 could enhance glycolysis in HCC, we knocked down *ZEB1* by short hairpin RNA (shRNA) in MHCC-97H and HCC-LM3 cells (both are hepatocellular carcinoma cell lines with high metastasis potential) and performed glucose uptake and lactate production assays. As shown in Figure 1A, ZEB1 was effectively knocked down, and both glucose uptake and lactate production were significantly decreased accordingly (Figure 1B-C). To exclude the off-target effect of shRNA, we constructed ZEB1 re-expression plasmid (designated by prefix “r”) which contains synonymous mutations in shRNA target sequence and is thus resistant to corresponding shRNA. Re-expression of ZEB1 restored the glycolytic flux in shRNA-expressing cells (Figure 1B-C). Consistently, downregulation of ZEB1 upregulated E-cadherin, a well characterized ZEB1 target gene whose expression is suppressed by ZEB1, confirming the knockdown (KD) of ZEB1. To clarify the regulatory effect of ZEB1 on the status of aerobic and anaerobic metabolism, we analyzed extracellular acidification rate (ECAR) and oxygen consumption rate (OCR) (Figure 1D-E). The steady state glycolysis flux, glycolytic capacity (Figure 1D, lower panels), basal respiration and ATP production (Figure 1E, lower panels) were attenuated in ZEB1 KD MHCC-97H cells, indicating that ZEB1 can promote glycolysis flux. In addition, overexpression of ZEB1 in both HUH-7 and Hep3B cells (Figure 1F) that express relatively lower levels of ZEB1 as compared with MHCC-97H and HCC-LM3 cells (Figure 2L), dramatically promoted glucose uptake (Figure 1G) and lactate production (Figure 1H). At the same time, the steady state glycolysis flux, glycolytic capacity, basal respiration and ATP production were also remarkably increased in HUH-7 cell by overexpression of ZEB1 (Figure 1I-J). In conclusion, ZEB1 is able to upregulate glycolytic rate and promote Warburg effect in HCC cell lines indeed.

### ZEB1 stimulates glycolysis by upregulating PFKM

To investigate how ZEB1 enhances glycolysis, we knocked down ZEB1 in MHCC-97H cells and detected the protein levels of glycolytic enzymes including PFKM, phosphofructokinase-1 liver type (PFKL), phosphofructokinase-1 platelet type (PFKP), hexokinase 1 (HK1), triose phosphate isomerase (TPI), aldolase (ALDOA) and glyceraldehydes-3-phosphate dehydrogenase (GAPDH), PKM2, as well as lactate dehydrogenase A (LDHA), a key enzyme required for Warburg effect by converting pyruvate to lactate. Among all the enzymes examined, PFKM protein level was exclusively decreased while the protein levels of the other two isoforms of PFK1, PFKL and PFKP, were not altered by ZEB1 KD (Figure 2A). Re-expression of ZEB1 restored PFKM protein level, suggesting that PFKM expression could be tightly regulated by ZEB1. To dissect whether downregulation of PFKM is responsible for diminished glycolytic flux caused by ZEB1 KD, we re-expressed PFKM in ZEB1 knockdown cells and detected glycolysis flux. Re-expression of either ZEB1 or endogenous level-comparable PFKM completely rescued the decrease of glucose uptake and lactate production as well as ECAR and OCR levels in ZEB1 KD MHCC-97H (Figure 2B-D, 2H-I) and HCC-LM3 cells (Figure S1A-C). Similarly, the promotion effect of overexpressed ZEB1 on glycolysis flux in HUH-7 (Figure 2E-G) and Hep3B cells (Figure S1D-F) was completely abolished by PFKM knockdown. These results indicate that ZEB1 stimulates glycolysis by upregulating PFKM. To further bolster this conclusion, we assessed the levels of glycolytic intermediate metabolites by employing liquid chromatography-mass spectrometry (LC-MS). As expected, the levels of three upstream intermediates of PFKM, fructose-6-phosphate (F6P, the substrate of PFKM) (Figure 2J), glucose-6-phosphate (G6P) and glucose-1-phosphate (G1P) (Figure S1G) were upregulated, on the contrary, three downstream intermediates, fructose-1,6-diphosphate (FBP, the production of PFKM) (Figure 2K), pyruvate and lactate (Figure S1I-G) were downregulated in ZEB1 KD MHCC-97H cells. Such alterations were further reversed by exogenous expression of either ZEB1 or PFKM, confirming our proposal that ZEB1 promotes glycolysis by upregulating PFKM. Interestingly, we observed that the abundance of 3-phosphoglyceric acid (3PG)/2-phosphoglyceric acid (2PG) was not significantly changed in ZEB1 KD cells (Figure S1H). It is worth noting that 3PG is a branch point of glycolysis. 3PG can also be used as a substrate for the synthesis of serine. One possible explanation is that ZEB1 may also be involved in the stimulation of serine synthesis and ZEB1 depletion simultaneously caused relative accumulation of 3PG, neutralizing its reduction resulting from glycolysis weakening. Consistently, ATP level was decreased in ZEB1 KD MHCC-97H and HCC-LM3 cells, and further reversed by re-expression of ZEB1 or PFKM (Figure S1K-L). Moreover, we detected ZEB1 and PFKM protein levels in a series of HCC cell lines and found a strong correlation between ZEB1 and PFKM protein levels (Figure 2L). Taken together, we suggest that ZEB1 stimulation of glycolysis is mediated by upregulation of PFKM.

### ZEB1 activates the transcription of PFKM through a non-classic binding sequence

**Figure 3:**
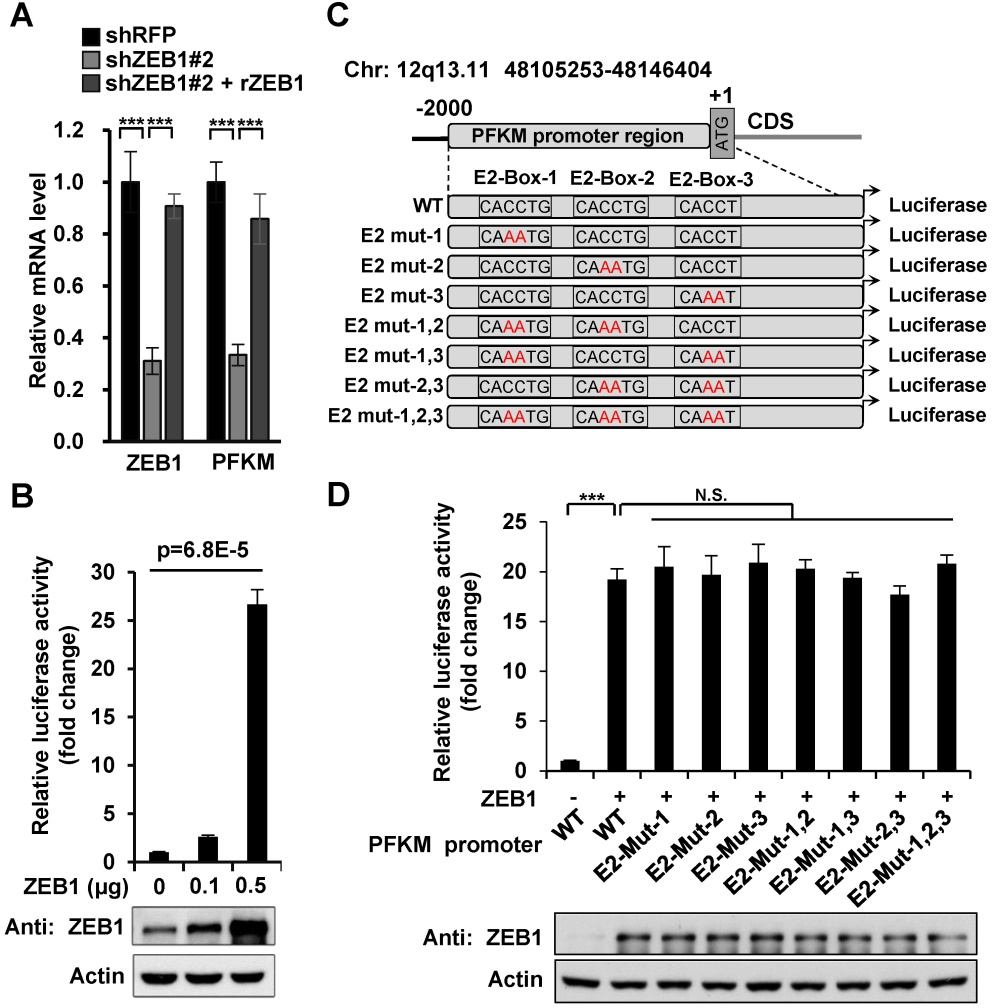
**Three E2-box-like sequences in the promoter region of PFKM are not required for its transactivation by ZEB1. (A)** MHCC-97H cell line was knocked down and further re-expressed for ZEB1. Then relative mRNA levels of ZEB1 and PFKM were determined using RT-qPCR. **(B)** HEK-293T cells were transfected with PFKM-Luc and increasing doses of ZEB1. 24 h post-transfection, luciferase activity was determined. The data are presented as means±SD of three independent experiments. Statistical analysis was performed by one-way ANOVA. **(C)** Schematic diagram showing wildtype PFKM promoter and its various mutants with three E2-box-like sequences mutated alone or in combinations. For each E2-box-like sequence, CA**CC**T(G) was mutated to CA**AA**T(G). **(D)** Luciferase reporter vectors carrying either wildtype PFKM promoter or any of its mutants was transfected into HEK-293T cells together with ZEB1. After 24 h of transfection, relative luciferase activity was determined and normalized to cells transfected with wildtype PFKM promoter alone (column 1). The data in (A) and (D) are shown as means±SD of three independent experiments (***P<0.001, N.S.: *P* ≥ 0.05, unpaired Student's *t* test).

Next, we explored how ZEB1 induces PFKM expression. Given that ZEB1 is a transcription factor, we examined the regulatory effect of ZEB1 on PFKM mRNA level. As expected, PFKM mRNA level was diminished dramatically in ZEB1 KD MHCC-97H cell line and such decrease was completely rescued by expression of ZEB1, suggesting that ZEB1 may be a transcriptional activator of PFKM (Figure 3A). To confirm this proposal, we created PFKM-Luc construct by cloning the putative PFKM promoter region (-2000 bp to +1 bp) into PGL2-basic vector and examined the regulatory effect of ZEB1 on it. As shown in Figure 3B, PFKM-Luc was greatly activated by expression of ZEB1 in a dose-dependent manner. Previous studies have clarified that the zinc finger clusters of ZEB1 can bind to the E2-box-like sequences CACCT(G) in the promoter region of its target genes and activate or repress the expression of these genes by recruiting co-activators or co-suppressors 4. There are three E2-box-like sequences in PFKM promoter regions (Figure 3C). To figure out whether ZEB1 binds to PFKM promoter through classic E2-box-like sequences, we created various mutants with these E2-box-like sequences mutated alone or in combinations. Surprisingly, none of all the mutants impaired the promoting effect of ZEB1 on PFKM-Luc (Figure 3D). These results indicated that ZEB1 may activate PFKM transcription by binding to PFKM promoter region through an unknown non-classic binding site.

**Figure 4:**
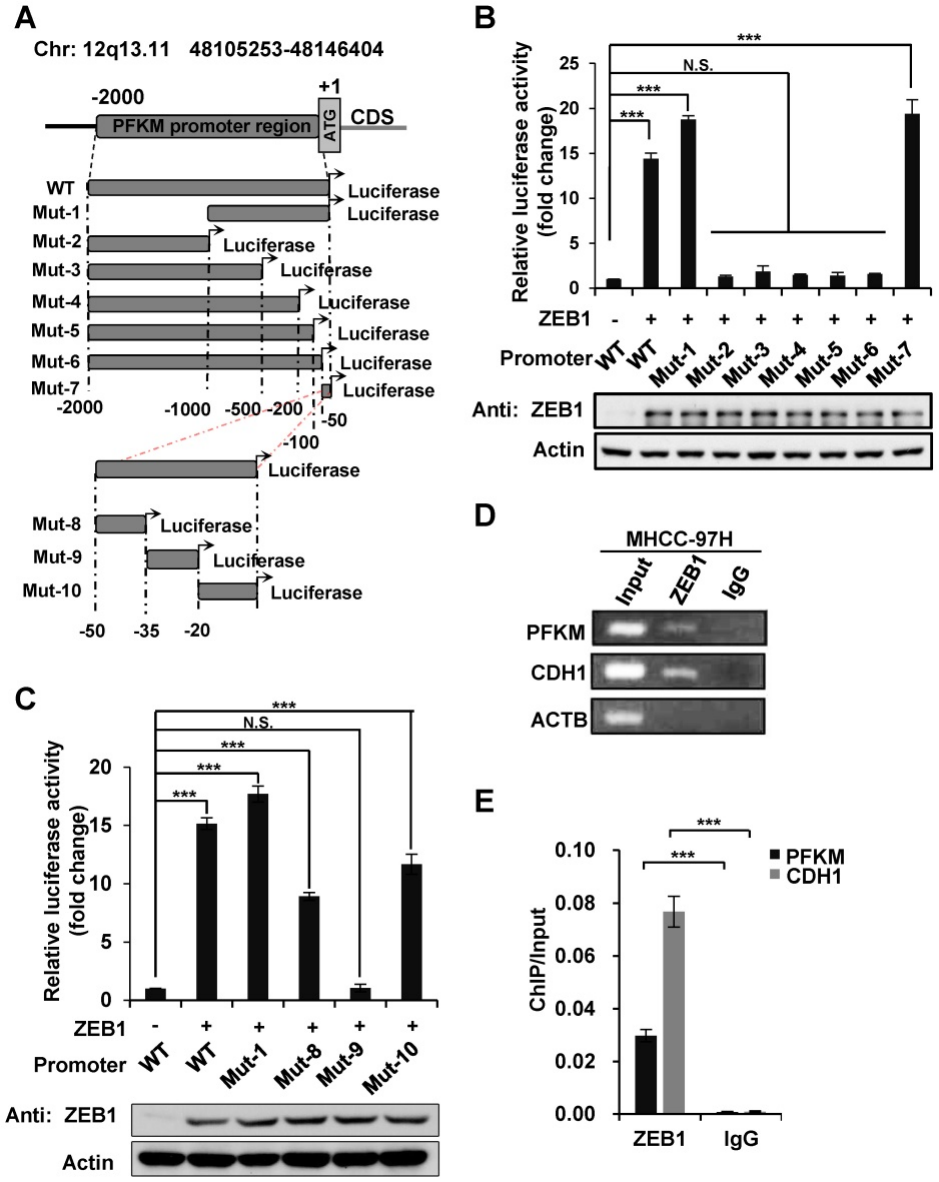
**ZEB1 activates PFKM transcription through a non-canonic binding sequence. (A)** Schematic diagram showing wildtype and truncated PFKM promoters. **(B-C)** Luciferase reporter vectors of wildtype PFKM promoter or its truncations were co-transfected with ZEB1 into HEK-293T cells, followed by determination of luciferase activity. The relative luciferase activity was normalized to cells transfected with wildtype PFKM promoter alone (column 1). **(D-E)** ChIP assay was performed in MHCC-97H cell line by using rabbit IgG and anti-ZEB1 antibody. CDH1 promoter serves as a positive control and ACTB promoter as a negative control (D). The data in (D) were quantified and normalized to input, and are presented as means±SD of three independent experiments (E) (***P<0.001, Student's *t* test).

To find out the ZEB1 binding site on PFKM promoter region, we constructed a series of truncated mutants of PFKM promoter and measured their luciferase activity in the presence of ZEB1 (Figure 4A). Mutants 2-6 which were all deleted of the 3' 50 bp sequences (designated as non-classic binding site, NCBS) lost response to ZEB1. Moreover, NCBS retained complete luciferase activity of full-length PFKM promoter. These results indicate that NCBS is either essential or sufficient for PFKM transcription activated by ZEB1. To further narrow down the exact ZEB1-binding site on NCBS, we divided NCBS sequences into three regions designated as Mut-8 (-50 bp to -35 bp), Mut-9 (-34 bp to -20 bp) and Mut-10 (-19 bp to +1 bp) (Figure 4A) and found that either Mut-8 or Mut-10 remained approximately half luciferase activity of 50 bp NCBS sequence, demonstrating that ZEB1 binds to PFKM promoter through Mut-8 plus Mut-10 sites residing in NCBS. Next, we performed chromatin immunoprecipitation (ChIP) assay to examine whether ZEB1 promotes PFKM expression by directly binding to its promoter region. Indeed, DNA fragments of PFKM promoter region, as determined by PCR-based amplification with primers specifically targeting the NCBS, were precipitated by ZEB1 antibody, but not IgG control (Figure 4D-E). In summary, ZEB1 activates the transcription of PFKM by directly binding to a non-classic ZEB-binding sequence on PFKM promoter.

### PFKM plays a key role in ZEB1-stimulated tumorigenesis and intrahepatic metastasis of HCC

**Figure 5:**
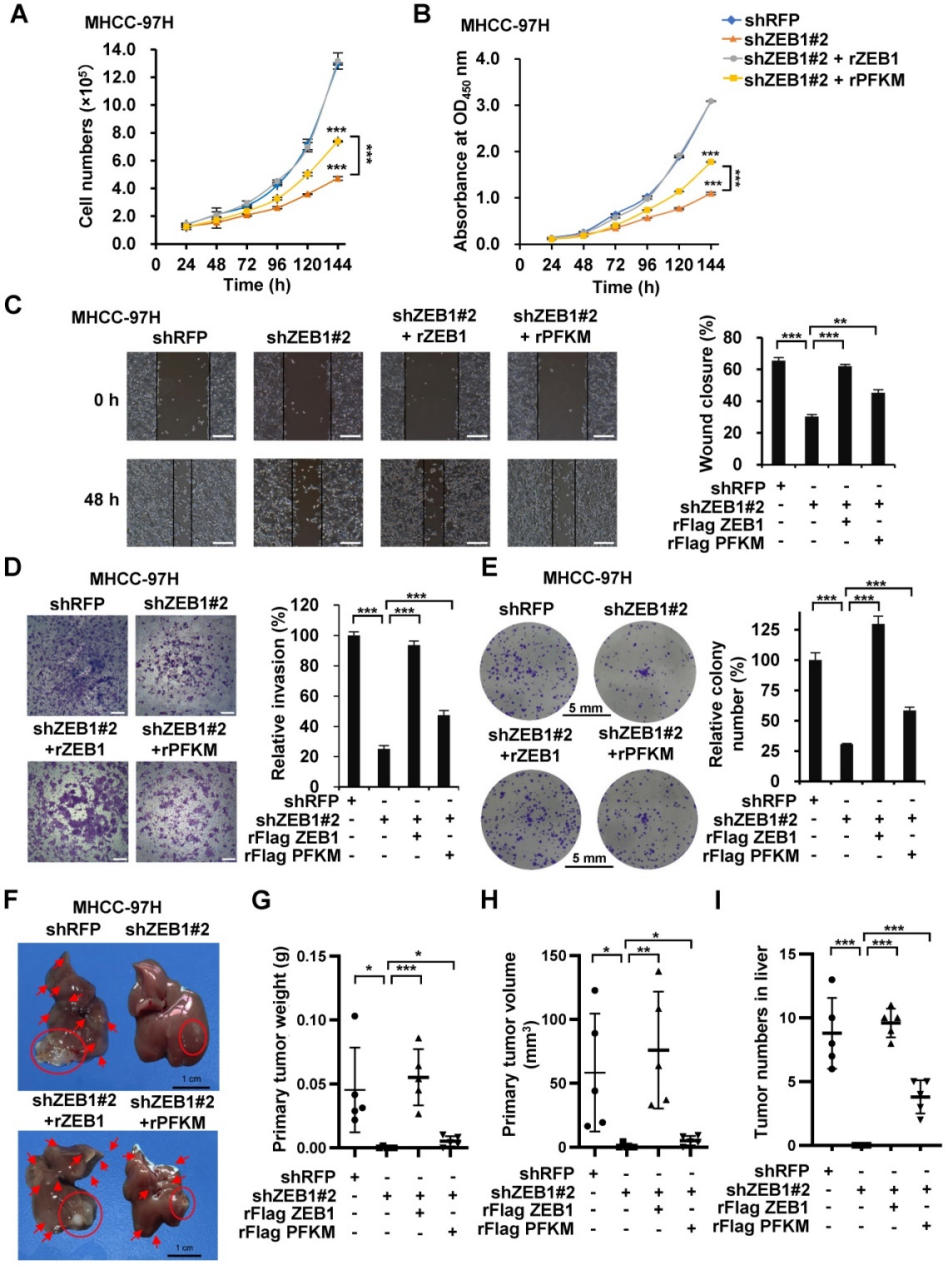
** PFKM plays a key role in ZEB1-stimulated tumorigenesis and intrahepatic metastasis of HCC. (A-B)** Cell proliferation were determined in ZEB1 KD MHCC-97H cells with or without further expression of ZEB1 and PFKM employing cell counting (A) and CCK-8 assays (B). **(C-I)** The same MHCC-97H cell lines as in (A) were performed for wound healing assays (C), transwell assays (D), colony formation assays (E) and orthotopic liver transplantation assays (F-I). Typical pictures showing mouse livers with tumor lesions (red circles indicate the primary tumor formed in the injection site and red arrows point to the intrahepatically metastatic tumor nodes on the surface of liver) (F). The primary tumor weight (G), primary tumor volume (H) and the number of metastatic tumor nodes (I) were determined. The scale bars in (C), (D), (E) and (F) represents 200 µm, 200 µm, 5 mm and 1 cm, individually. The data in (A, C-E) are shown as means±SD of three independent experiments, in (B) are shown as means±SEM of three independent experiments in quadruplication (*P<0.05, **P<0.01, ***P<0.001). The data in (G-I) are means±SD (n=5) (*P<0.05, **P<0.01, ***P<0.001). The data in this Figure are analyzed statistically using unpaired Student's *t* test where necessary.

ZEB1 is one of the most important drivers of EMT and cancer progression. Our results above show that ZEB1 upregulates PFKM and enhances glycolysis. We spontaneously want to know whether ZEB1-PFKM axis contributes to tumor progression. In line with above study, the cell proliferation rate was markedly retarded by ZEB1 knockdown in MHCC-97H (Figure 5A-B) and HCC-LM3 (Figure S2A-B) cells. Such retardation was alleviated by overexpression of PFKM, indicating that ZEB1 promotes cell proliferation at least partially via upregulating PFKM expression. Since ZEB1 is an important regulator of EMT and cancer metastasis, we explored whether the ZEB1-PFKM axis also functions in HCC cell migration, invasion and metastasis. The wound healing and transwell assays showed that ZEB1 knockdown remarkably weakened the migration and invasion capabilities of MHCC-97H (Figure 5C-D) and HCC-LM3 (Figure S2C-D) cells, and such weakening was restored by further expression of PFKM. We also performed soft-agar colony formation assay and observed that overexpression of PFKM could rescue the attenuation of anchorage-independent growth caused by ZEB1 KD in both MHCC-97H (Figure 5E) and HCC-LM3 cells (Figure S2E). These observations demonstrate that upregulation of PFKM expression is crucial for ZEB1 to promote cell migration and invasion. To answer whether ZEB1-PFKM axis contributes to tumor progression *in vivo*, we performed *in vivo* tumor formation experiment by injecting MHCC-97H cells into the same site of the left liver lobe of nude mice to develop orthotopic liver tumor. After 6 weeks of the injection, livers were isolated from each group of mice and the weight and size of primary tumor in the injection site were determined to indicate the tumorigenic ability of each cell lines. Simultaneously, the number of intrahepatically metastatic tumors excluding the primary tumor was counted to reflect the metastatic capability of injected cell lines. As shown in Figure 5F-I, knockdown of ZEB1 in MHCC-97H cells significantly decreased the weight and size of primary tumor and the number of metastatic tumors on the surface of liver. Importantly, such decrease could be partially restored by further expression of PFKM. These data demonstrate that PFKM plays an important role in ZEB1-stimulated tumorigenesis and intrahepatic metastasis of HCC.

### Both ZEB1 and PFKM are upregulated in HCC and correlated with poor prognosis

**Figure 6:**
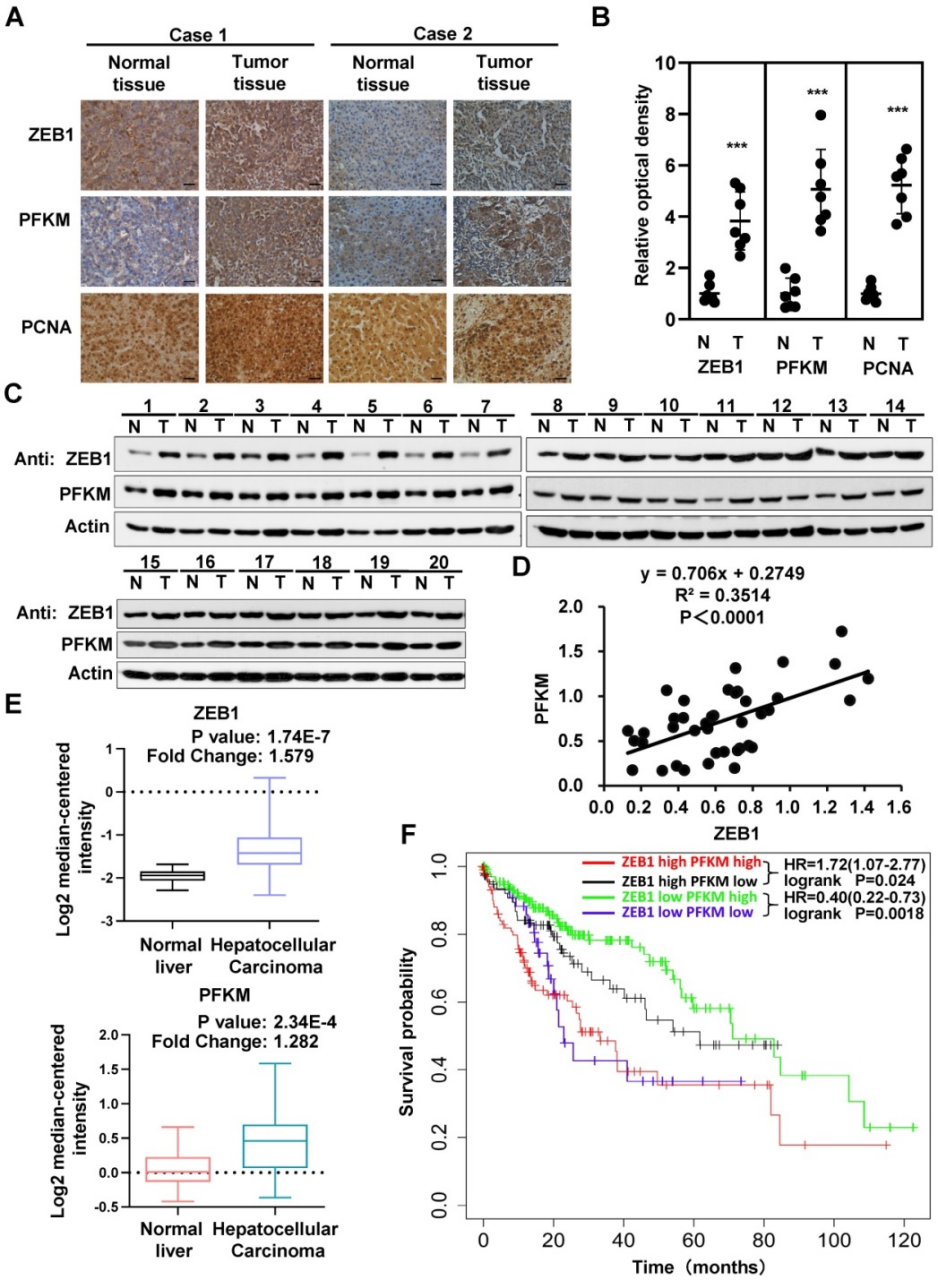
**Interrelated high expression of ZEB1 and PFKM is correlated with poor prognosis of HCC. (A-B)** Representative pictures (A) and relative optical density of IHC staining of ZEB1, PFKM and PCNA in HCC tissues and the corresponding adjacent normal tissues (B). The scale bars represent 100 µm. The data are shown as means±SD (n=7, ***P <0.001, Student's *t* test). **(C-D)** The protein levels of ZEB1 and PFKM in HCC tissues and the corresponding adjacent normal tissues (n=20) were determined using Western blot (C), followed by analysis of their correlation (D). **(E)** The comparison of either ZEB1 (upper panel) or PFKM (lower panel) expression between normal liver tissues and HCC tissues. Data are publicly available in Oncomine (the MAS liver). **(F)** The Kaplan-Meier curves of overall survival basic relating to ZEB1-PFKM axis in HCC. Data are publicly available in the Kaplan-Meier Plotter.

The above data clearly establish that PFKM plays a key role in the oncogenicity of ZEB1. We thus examined the expression and correlation of ZEB1 and PFKM in clinical HCC specimens. The expression levels of ZEB1 and PFKM are markedly higher in HCC tissues than in corresponding adjacent normal tissues and show close correlation as determined by IHC staining and Western blot analysis (Figure 6A-D). The Mas liver database from the Oncomine also indicates that the expression levels of ZEB1 and PFKM are higher in HCC tissue than in normal liver tissue (Figure 6E). Furthermore, the data from Kaplan-Meier Plotter shows that in ZEB1 high cohort, high expression of PFKM is associated with poor prognosis. While in ZEB1 low cohort, lower expression of PFKM predicts a worse prognosis. These observations suggest that higher correlation between ZEB1 and PFKM expression reflects poor prognosis of HCC. In conclusion, ZEB1 and PFKM are upregulated in HCC and their good correlation predicts poor prognosis, reinforcing our proposal that ZEB1 promotes progression of HCC partially by activating PFKM transcription and resultant augmentation of glycolysis.

## Discussion

In recent years, growing evidences have indicated that ZEB1 (also known as TCF8, AREB6, ZFHEP, ZFHX1α, BZP, NIL-2α, deltaEF1) plays vital roles in EMT and cancer metastasis and its higher expression correlates with poor clinical outcomes in HCC patients 16-18. The mechanism by which ZEB1 promotes EMT has been widely investigated and clarified. As a transcription factor of Zinc finger E-box-binding homeobox family, ZEB1 can bind to E2-box-like CACCT(G) sequences in the promoter region of E-cadherin, and subsequently suppress its expression. Since E-cadherin plays a key role in maintaining the epithelial phenotype 5, its suppression eventually results in EMT, a key biological event for metastasis of tumor cell. ZEB1 was also reported to activate the transcription of glucose transporter 3 (GLUT3) 19 and suppress the transcription of Fructose-1,6-bisphosphatase 1 (FBP1) 20, a key enzyme in gluconeogenesis. However, whether ZEB1 can stimulate Warburg effect by transcriptionally activating any of the ten glycolytic enzymes and consequently contribute to tumorigenesis and metastasis is largely unknown.

Based on our previous study showing that ZEB1 was excessively expressed in HCC and its high expression was closely correlated with metastasis and recurrence of HCC, we examined its possible regulatory role on glycolytic enzymes, considering the importance of Warburg effect as a hallmark of tumor metabolism in tumorigenesis and progression. Firstly, knockdown of ZEB1 significantly decreased the protein level of PFKM and aerobic glycolysis as indicated by the drop of glucose uptake, lactate production and ECAR level, meanwhile, such decrease was largely rescued by exogenous expression of PFKM, raising the possibility that ZEB1 stimulates glycolysis via orchestrating PFKM expression. Secondly, we constructed the luciferase reporter vector containing the putatively full promoter region (-2000 bp to +1 bp) of PFKM and a series of its deletions. Luciferase assays indicated that two regions, Mut-8 (-50 bp to -35 bp) and Mut-10 (-19 bp to +1 bp) that reside in the NCBS sequence of PFKM promoter is required for ZEB1 activation. This observation was confirmed by ChIP assay showing that NCBS was precipitated by ZEB1. Up to this point, we provide convincing evidence that ZEB1 can activate PFKM transcription by directly binding to NCBS in its promoter region. Thirdly, we made great effort to find the biological significance of such regulation. Silencing of ZEB1 in MHCC-97H cell leads to impaired proliferation and invasion and such impairments are mainly resulted from the downregulation of PFKM expression. Moreover, *in-situ* HCC xenograft assays demonstrated that ZEB1-PFKM-glycolysis regulatory axis is crucial for tumorigenesis and intrahepatic metastasis of HCC, consistent with the studies from TCGA database. Our study thus reveals a novel aspect of ZEB1 in promoting HCC by activating PFKM and glycolysis. Importantly, our proposal is supported by other lines of evidence. It has been reported that higher expression of PFKM is closely correlated with the increased glycolysis in human lung adenocarcinoma 21. Furthermore, a recent research announced that suppression of FBP1 transcription by ZEB1 is a critical oncogenic event in lung cancer progression 20. As a key enzyme in gluconeogenesis, FBP1 reverses the reaction catalyzed by PFKM. That is to say, inhibition of FBP1 indirectly increases the glycolytic step catalyzed by PFKM. It is possible that ZEB1 may stimulate glycolysis by simultaneously activating PFKM and inhibiting FBP1 transcription in HCC, and the later postulation should be further investigated in HCC in the future.

As a transcriptional factor, ZEB1 ordinarily suppresses or activates the transcription of its target genes by binding to the classic E2-box-like sequences CACCT(G) in the promoter region of these genes. Extraordinarily, to the case of PFKM, in spite of three putative classic E2-box-like sequences (E2-Box-1: CACCTG; E2-Box-2: CACCTG; E2-Box-3: CACCT) in its promoter region, ZEB1 activates the transcription of PFKM by associating exclusively with NCBS, a 3′ 50 bp sequence in its promoter. One possible explanation for our observation is that there may exit at least a co-transcriptional factor for ZEB1 activation of PFKM and this factor may influence the DNA binding property and priority of ZEB1. Our finding thus increases the complexity of ZEB1 as a transcriptional factor in regulating different target genes.

Taken all together, we identified a novel mechanism of ZEB1 in promoting HCC by activating the transcription of PFKM, establishing the direct link of ZEB1 to the promotion of glycolysis and Warburg effect. Thus, strategy that disrupts the regulatory effect of ZEB1 on PFKM should be developed as a treatment to HCC and other cancers with excessive expression of PFKM driven by ZEB1.

## Supplementary Material

Supplementary figures and tables.

## Abbreviations

ZEB1: Zinc finger E-box-binding homeobox 1
EMT: Epithelial-mesenchymal transition
PFKM: muscle isoform of phosphofructokinase-1
shRNA: short hairpin RNA
NCBS: non-classic binding site
ChIP: chromatin immunoprecipitation
FBP1: Fructose-1,6-bisphosphatase 1
