# Supplementary material for: ZEB1 enhances Warburg effect to facilitate tumorigenesis and metastasis of HCC by transcriptionally activating PFKM
Source: Theranostics. 2021 Apr 3;11(12):5926–38. doi: 10.7150/thno.56490 (PMC8058737; doi:10.7150/thno.56490)
Supplement: Supplementary file 1 — Supplementary figures and tables. [file thnov11p5926s1.pdf]

Key Resource Table:
Table S1 Antibodies for Western blot and IHC

Antibody	Source	Catalog number	Application(dilution)
ZEB1	Proteintech	Cat #21544-1-AP	WB (1:1000), IHC (1:200)
PFKM	Proteintech	Cat #55028-1-AP	WB (1:1000), IHC (1:200)
PFKL	Proteintech	Cat #15652-1-AP	WB (1:1000)
PFKP	Proteintech	Cat #13389-1-AP	WB (1:1000)
E-Cadherin	Proteintech	Cat #20874-1-AP	WB (1:5000)
Actin	Proteintech	Cat # AC-15	WB (1:5000)
HK1	Proteintech	Cat # 66974-1-Ig	WB (1:1000)
ALDOA	Proteintech	Cat #11217-1-AP	WB (1:1000)
TPI	Proteintech	Cat #10713-1-AP	WB (1:1000)
ENO1	Proteintech	Cat #11204-1-AP	WB (1:500)
GAPDH	Proteintech	Cat #60004-1-Ig	WB (1:5000)
G6PD	Proteintech	Cat #25413-1-AP	WB (1:1000)
LDHA	Proteintech	Cat #19987-1-AP	WB (1:5000)
PCNA	Proteintech	Cat # 10205-2-AP	IHC (1:200)

Table S2 Primers used in this study

Table S2-1 Primers for over expression plasmid constructions:

Gene	sequence
ZEB1	F: 5′-ATGGCGGATGGCCCCAG-3′
	R: 5′-GGCTTCATTTGTCTTTTCTTC-3′
ZEB1	F: 5′-AGTGGCGGTAGACGGCAACGTA-3′
(RNAi resistance)	R: 5′-CTTATTACGTTGCCGTCTAC-3′
PFKM	F: 5′- AGAGAATTCGGATCC-3′
	R: 5′- CTTCCATGGCTCGAG-3′

Table S2-2 Primers for PLL3.7 shRNA constructions:

Gene	Target sequence
shRFP	5′-GAAATCCACCCGGGGCGCCC-3′
shPFKM#1	5′-CCTCCAGAAAGCAGGTAAGATT-3′
shZEB1#2	5′-GGTAGATGGTAATGTAATAT-3′

Table S2-3 Primers for promoter constructions:

Name	sequence
Full length	F: 5′-GCGTGCTAGCTCGAGATTTGGCCTACAGGCTGTTG-3′
	R: 5′-CGGAATGCCAAGCTTCATGATCCACTCTAGAATTTAAG-3′
E2 mut -1	F: 5′-AAATAACAAATTTTAGCCTGAAAAAC-3′
	R: 5′-TAAAATTTGTTATTTAAATTTATCTCC-3′

**E2 mut -2**	**F: 5′-CAGTTCAAATGGAAAATGGCAATG-3′**
	**R: 5′-ATTTTCCATTTGAACTGCTTACTAC-3′**
**E2 mut -3**	**F: 5′-TTCAAATGACAATGGAGCAGAGAAT-3′**
	**R: 5′-ATTGTCATTTGAACATTACTTTGC-3′**
**Mut-1**	**F: 5′-GCGTGCTAGCTCGAGGGGATGGATAAAGGAAGATAATAAG-3′**
	**R: 5′-CGGAATGCCAAGCTTCATGATCCACTCTAGAATTTAAG-3′**
**Mut-2**	**F: 5′-GCGTGCTAGCTCGAGATTTGGCCTACAGGCTGTTG-3′**
	**R: 5′-CGGAATGCCAAGCTTCTCCTTTCTATCCTCATTGC-3′**
**Mut-3**	**F: 5′-GCGTGCTAGCTCGAG ATTTGGCCTACAGGCTGTTG-3′**
	**R: 5′-CGGAATGCCAAGCTTGTGTTATGGAATTGTGCTATTG-3′**
**Mut-4**	**F: 5′-GCGTGCTAGCTCGAG ATTTGGCCTACAGGCTGTTG-3′**
	**R: 5′-CGGAATGCCAAGCTTAAATGTCATGTAATCAAGCCC-3′**
**Mut-5**	**F: 5′-GCGTGCTAGCTCGAG ATTTGGCCTACAGGCTGTTG-3′**
	**R: 5′-CGGAATGCCAAGCTTCTCAGTCAGGCTCTCCCAC-3′**
**Mut-6**	**F: 5′-GCGTGCTAGCTCGAGATTTGGCCTACAGGCTGTTG-3′**
	**R: 5′-CGGAATGCCAAGCTTATCAAGATGCCACTAGCTAAAG-3′**
**Mut-7**	**F: 5′-GCGTGCTAGCTCGAGTCCTGCTGTGTCTTAACTG-3′**
	**R: 5′-CGGAATGCCAAGCTTCATGATCCACTCTAGAATTTAAG-3′**
**Mut-8**	**F: 5′-TCGAATTCTAGAGTGGATC-3′**
	**R: 5′-AGCTGATCCACTCTAGAAT-3′**
**Mut-9**	**F: 5′-TCGATTAAGACAATGGTCA-3′**
	**R: 5′-AGCTTTAAGACAATGGTCA-3′**

**Mut-10**	**F: 5′-TCGAGATTCCTGCTGTGTCTTAAC-3′**
	**R: 5′-AGCTGTTAAGACACAGCAGGAATC-3′**

Table S2-4 Primers for RT-qPCR:

Gene	sequence
**ACTB**	**F: 5′-TCCATCATGAAGTGTGACGT-3′**
	**R: 5′-TACTCCTGCTTGCTGATCCAC-3′**
**ZEB1**	**F: 5′-CGAACCCGCGGCGCAATA-3′**
	**R: 5′-CCAGCAGTTCTTAGCATTCC-3′**
**PFKM**	**F: 5′-GACCCGTGGTTCTCGTCTC-3′**
	**R: 5′-AAAGGCTGATGGCGTCCC-3′**

Table S2-5 Primers for ChIP:

Gene	sequence
**PFKM**	**F: 5′-AGCTAGTGGCATCTTGATTCATGAAGCAAAGGGAAGAGTG-3′**
	**R: 5′-CTTGGGCATCTCCACCAGAGGACGGCAGCTTCCCCG-3′**
**CDH1**	**F: 5′-GGCCGGCAGGTGAACCCTCA-3′**
	**R: 5′-GGGCTGGAGTCTGAACTGA-3′**
**ACTB**	**F: 5′-GTGCAAG AATCCCGTATA-3′**
	**R: 5′-TTGAGTCCAGGAGTTTGA-3′**

Supplementary Figures and Figure legends:

Figure S1 (related to Figure 2):

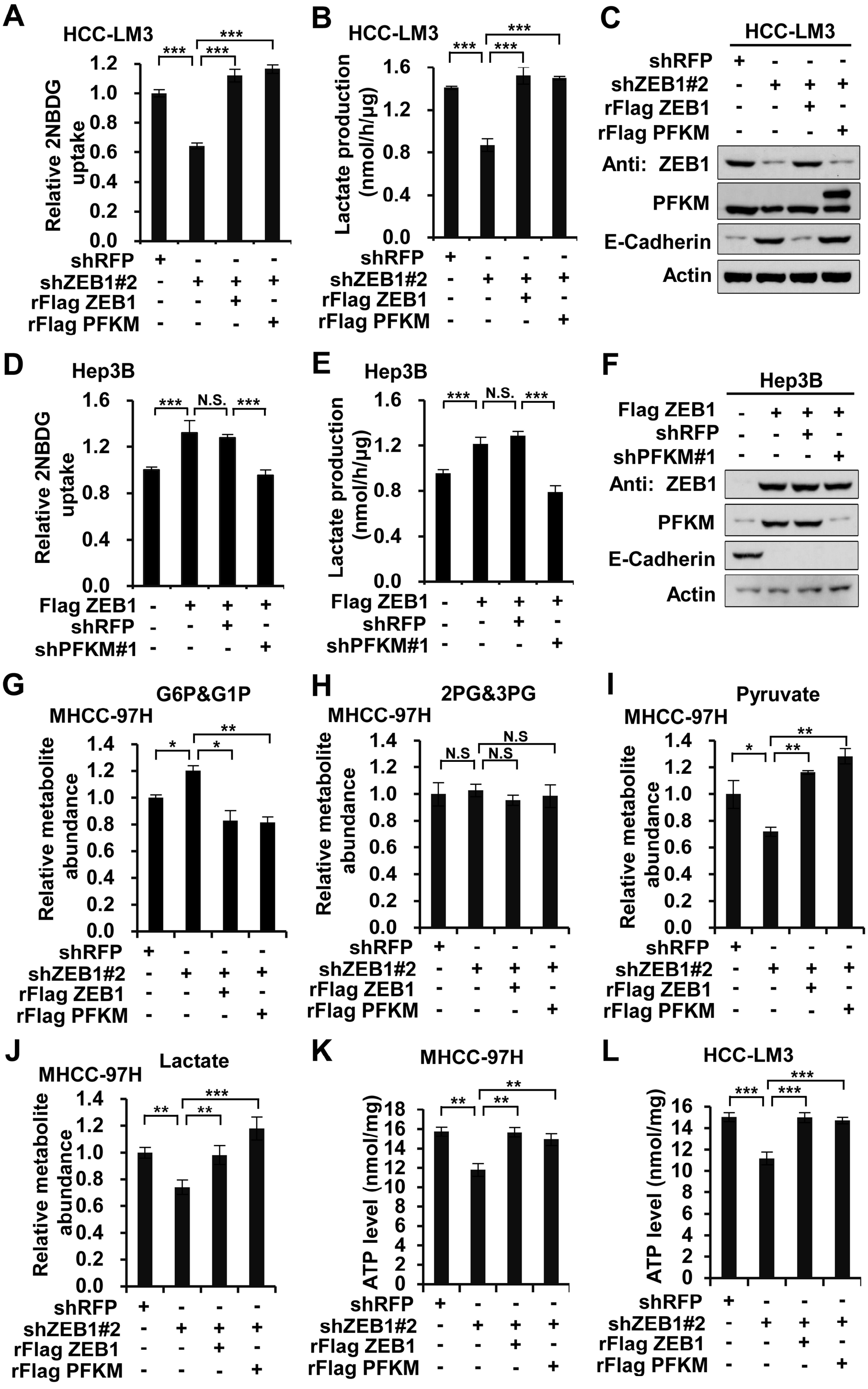

Figure S1 ZEB1 stimulates glycolysis by upregulating PFKM. **(A-C)** HCC-LM3 cells were knocked down for ZEB1 and further expressed for exogenous ZEB1 or PFKM, followed by determination of expression levels of glucose uptake (A), lactate

production (B) and indicated proteins (C). **(D-F)** Hep3B cells with low endogenous expression of ZEB1 was overexpressed for Flag-tagged ZEB1 and further knocked down for PFKM, followed by measurement of glucose uptake (D), lactate production (E) and protein levels (F). **(G-K)** Relative levels of G6P&G1P (G), 2PG&3PG (H), pyruvate (I), lactate(J) and ATP in the same MHCC-97H cell lines as in Figure 2B were measured using LC-MS. **(L)** The same HCC-LM3 cell lines as in (A) were measured for ATP level. All of the statistical data in this Figure are shown as means±SD of three independent experiments (Student's *t* test, * $P < 0.05$, ** $P < 0.01$, *** $P < 0.001$, N.S.: $P \geq 0.05$).

Figure S2 (related to Figure 5):

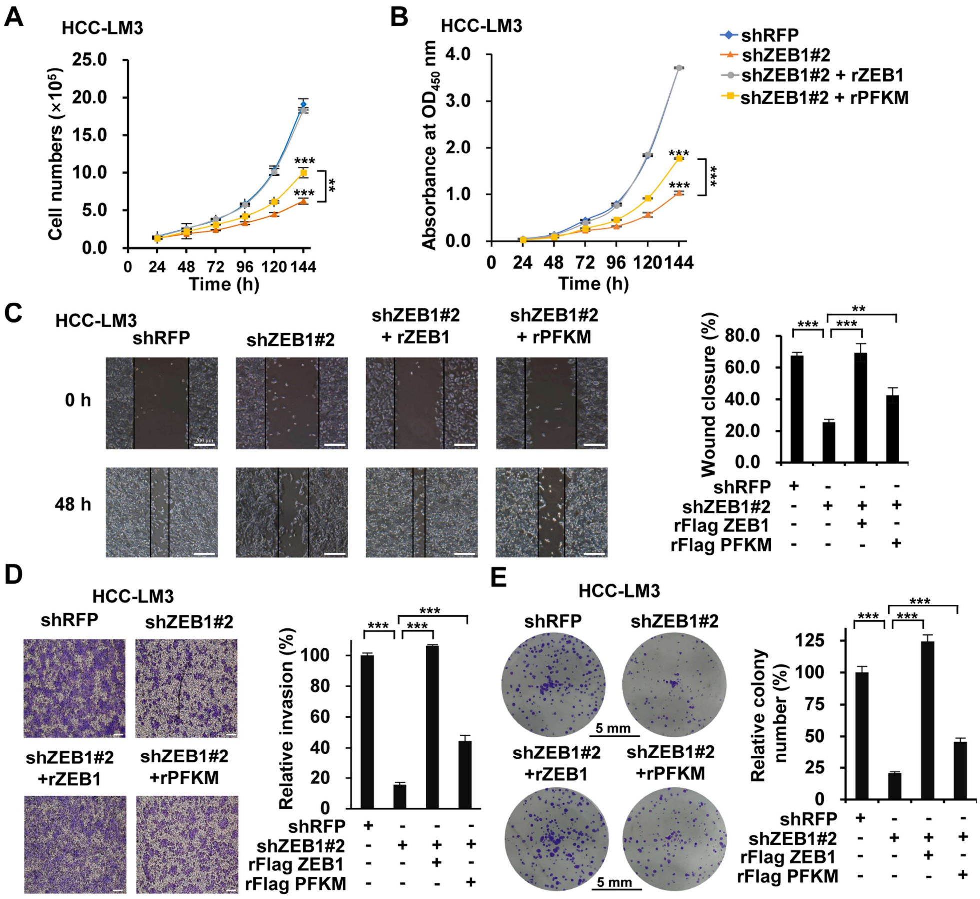

Figure S2 PFKM plays a key role in ZEB1-stimulated proliferation, migration, invasion and anchorage-independent growth of HCC cells. (A-B) Cell proliferation

were determined in ZEB1 knockdown HCC-LM3 cells with or without further expression of ZEB1 or PFKM by employing cell counting (A) and CCK-8 assays (B). **(C-E)** The same cell lines as in (A) were performed for wound healing assays (C), transwell assays (D) and colony formation assays (E). The scale bars in (C), (D) and (E) represents 200 μm, 200 μm and 5 mm, individually. The data in (A, C-E) are shown as means±SD of three independent experiments, in (B) are shown as means±SEM of three independent experiments in quadruplication (** $P < 0.01$, *** $P < 0.001$, unpaired Student's *t* test).
